# O048: OXA-181- carbapenemase producing Klebsiella pneumoniae: an emerging threat? The first reported nosocomial outbreak in Singapore

**DOI:** 10.1186/2047-2994-2-S1-O48

**Published:** 2013-06-20

**Authors:** L Alenton, MY Hsann, P Tambyah, R Lin, KS Ng, S Rethanam, L Poh, SL Soong, SK Pada

**Affiliations:** 1 Infection Control, Alexandra Hospital, Jurong Health Services, Singapore; 2 Epidemiology, Alexandra Hospital, Jurong Health Services, Singapore; 3 Medicine, National University Health System, Singapore; 4 Laboratory Medicine, National University Health System, Singapore; 5 Medicine, Alexandra Hospital, Jurong Health Services, Singapore, Singapore

## Introduction

Carbapenem-resistant Enterobacteriaceae (CRE) are an emerging global threat. Most outbreaks have been NDM-1 or KPC. We describe an outbreak of OXA-181-producing *Klebsiella pneumoniae* in a 275 bedded acute general hospital in Singapore.

## Methods

A patient who was managed in a 24 bedded, Male, Geriatric, Transition & Rehabilitation unit had a blood culture positive for Klebsiella pneumoniae resistant to Imipenem and Meropenem (MIC 6.000mg/L and 24.000mg/L ) on 24 January 2013. Contact tracing with rectal swabs was done for all patients in the same cubicle and then ward. The ward and adjacent gym were closed, CRE patients were isolated in single rooms, patient areas and bathroom facilities were cleaned and disinfected. Hand hygiene and isolation education were reinforced to all healthcare staff. All patients were swabbed during outpatient follow up and during readmission. The unit was reopened after 12 days when no new cases of CRE were identified from remaining patient contacts. All positive CRE isolates were sent to a reference laboratory for further typing.

## Results

Two of five (40%) patients who had stayed in the same 6- bedded cubicle as the index case were found to have CRE. Three of 9 (33%) of the rest of the ward patients were CRE positive. A further two contacts had CRE detected in urine specimens 4 days and a week later. Overall, the attack rate for patients in the same cubicle was 3/6 (50%) and ward was 3/9 (33%). A screen of the adjacent ward did not identify any CRE patients among 18screened. All isolates were identical by Pulsed Field Gel Electrophoresis bearing *bla*_OXA-181._

## Conclusion

This strain of OXA-181-producing *Klebsiella pneumonia* has clear outbreak potential. Prompt action with strong multidisciplinary support and the ability to close the affected ward enabled us to contain this outbreak. Clinicians worldwide need to be alert to the threat of this emerging nosocomial pathogen.

## Disclosure of interest

None declared.

